# A rare variant of the superficial ulnar artery, and its clinical implications: a case report

**DOI:** 10.1186/1752-1947-1-128

**Published:** 2007-11-07

**Authors:** Kithsiri J Senanayake, Sujatha Salgado, Manjula J Rathnayake, Ranil Fernando, Kosala Somarathne

**Affiliations:** 1 Department of Anatomy, Faculty of medicine, Ragama, Sri Lanka; 2 Department of Surgery, Faculty of medicine, Ragama, Sri Lanka; 3 Professorial surgical unit teaching hospital, Peradeniya, Sri Lanka

## Abstract

The superficial ulnar artery is a rare variation of the upper limb arterial system that arises from the brachial or axillary artery and runs superficial to the muscles arising from the medial epicondyle [1-3]. The incidence is about 0.7 to 7% [1,4,5]. In our routine dissections we found a superficial ulnar artery, which crossed the cubital fossa superficial to the bicipital aponeurosis making it highly vulnerable to intra-arterial injection. This is a rare variation that every medical and nursing staff member should know about.

## Case presentation

During the dissection of cadavers to prepare prosected specimens for the medical students of the Department of Anatomy, Faculty of Medicine, Ragama, Sri Lanaka, an unusual branch of the brachial artery was found in one cadaver. Thereafter the arterial systems of both upper limbs were traced carefully.

**Figure 1:**
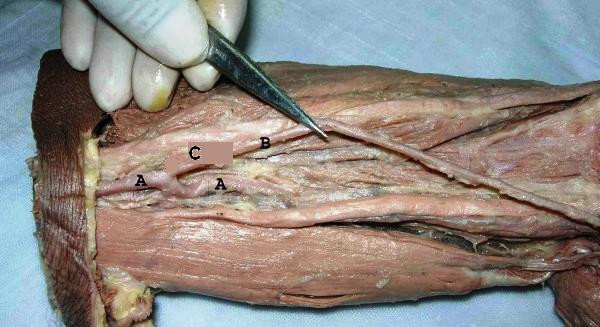
The origin of the superficial ulnar artery (C) from upper one third of the brachial artery (A). Superficial ulnar artery (B).

**Figure 2:**
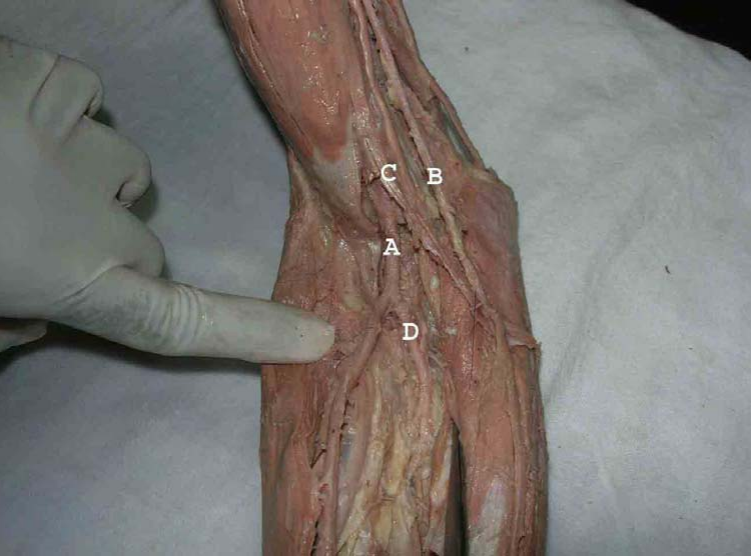
The superficial ulnar artery (B) at the cubital fossa where it runs superficial to the bicipital aponeurosis (C) The brachial artery (A) divides in the cubital fossa into the common interosseous artery (D) and radial artery. Note, the radial artery is bigger than the superficial ulnar artery.

The ulnar artery was arising from the medial side of the upper one-third of thebrachial artery [Fig. 1C]. It was descending deep to the deep fascia. One inch above the elbow, piercing the deep fascia it came to lie superficial to the bicipital aponeurosis [Fig. 2]. At the cubital fossa the artery crossed from the medial to the lateral side and passed superficial to the muscles arising from the medial epicondyle (Fig 2). At the distal third of the forearm the course of the ulnar artery was normal and passed superficial to the flexor retinaculum to form the superficial palmar arch as usual. It gave off a branch at mid arm level that passed behind the medial epicondyle. There were no branches throughout its course in the forearm and it was running superficial to the deep fascia. The brachial artery was divided into the radial artery and the common interosseous artery at the cubital fossa [Fig. 2]. The radial artery was larger and took a normal course (Fig. 2). The common interosseous artery passed deep to the pronator teres muscle giving off median, posterior interosseous and anterior interosseous branches. The arterial pattern of the right upper limb was normal.

## Discussion

Variations of the upper limb arterial system are well documented [1,4,5]. The presence of a superficial ulnar artery seems to be a rare variation with an incidence of 0.7%–7% in the literature [1,4,5]. Its clinical importance should not be underestimated as several cases of intra-arterial injection of drugs and subsequent amputations have been reported [6,7].

The ulnar artery was found to deviate from its usual mode of origin in one in thirteen cases; frequently arising from the lower end of the brachial artery [2]. In case of high origin it descends over the muscles arising from the medial epicondyle deep to the deep fascia [1,8]. On a few occasions the superficial ulnar artery was reported bilaterally [9,10]. Bilateral cases are more often seen in females [1]. The superficial ulnar artery may arise from any part of the brachial or axillary artery and frequencies are summarised in Table 1[1].

**Table 1:** Origins of the superficial ulnar artery [1]

	No of cases	Axillary artery	Brachial artery	Upper 1/3	Middle 1/3	Lower 1/3
Quain (1844)	31	8	23	6	1	16
Gruber (1867)	20	4	16			
Breme (1899)	7	1	6	1	0	5
Muller (1903)	2	1	1	0	0	1
McCormack (1953)	17	7	10			
FuB et al. (1985)	3	2	2	0	0	0
Rpdriguez-Baeza (1995)	8	2	6	3	2	1
Rodriguez M (2000)	16	4	12	6	0	3
Total	104	28(26.9%)	76(73.1%)	16(32%)	3 (6%)	26(52%)

The ulnar artery that we found was somewhat similar to the variation described by Panikar et al. (2003) and the same pattern as that named as the superficial brachio-ulnar artery by Rodriguez et al. (2001). But in our case the artery was superficial to the bicipital aponeurosis.

## Embryological basis

The 7th intersegmental artery forms the axis artery of the upper limb. The axis artery gives rise to the axillary, brachial and interosseous arteries. Other branches are added subsequently to the axis artery. First is the median artery. The ulnar and the radial arteries arise from the axis artery later. Because of this temporal succession of emergence of principle arteries, anomalies of forearm vasculature occur[3]. This sprouting theory is greatly challenged by the study of Rodriguez et al 2001. Their findings suggest that the arterial pattern of the upper limb develops from an initial capillary plexus by a proximal and distal differentiation, due to maintenance, enlargement and differentiation of certain capillary vessels, and the regression of others [11]. The embryological basis of arterial variations could be described by modification of normal capillary maintenance and regression [11].

## Clinical significance

Superficial position of the ulnar artery makes it more vulnerable to trauma and thus haemorrhages. In our described case the ulnar artery was superficial to the bicipital aponeurosis and in a clinical setting such a variant could easily be mistaken for a vein. Such misinterpretation could lead to intra-arterial injection and difficulties in angiographic procedures [5]. It also has would have a higher risk of getting damaged during forearm surgery. Demonstration of patency of the ulnar artery is very important before raising a free radial forearm flap, and can be established by Doppler flow meter. After such a flap forearm blood supply would depend entirely on the patent ulnar artery. Hence any abnormal positions or divisions of the ulnar artery are important to identify before such surgery [12]. The importance of this variant is growing with the increasing use of radial arteries as conduits in coronary bypass [13].

## Conclusion

Occurrence of the superficial ulnar artery is a rare variation. But the presence of it is very important clinically. The knowledge of such variation is important; not only for surgeons but also for other medical and nursing staff because intravascular canulations are commonly performed by them. We recommend palpating for a superficial pulse over the canulation site before such a procedure. This simple procedure will probably minimize the risk of damaging an artery and subsequent bleeding.

## Consent

Informed written consent for photographing and publishing of this case report was taken from the son of the deceased.

## Competing interests

The author(s) declare that they have no competing interests.

## Authors' contributions

KJ was responsible for the study conception and design, writing the manuscript, dissection and literature review. SS was responsible for the literature review, dissection and proofreading the manuscript. MJ was responsible for the literature review and helping to write the manuscript. KS was responsible for the literature review and helped to write the manuscript regarding the surgical aspects. RF was responsible for the literature review, helping to write the manuscript regarding the surgical aspects. All authors read and approved the final manuscript
